# Is the Mouse a Good Model of Human PPARγ-Related Metabolic Diseases?

**DOI:** 10.3390/ijms17081236

**Published:** 2016-07-30

**Authors:** Attila Pap, Ixchelt Cuaranta-Monroy, Matthew Peloquin, Laszlo Nagy

**Affiliations:** 1 Department of Biochemistry and Molecular Biology, Faculty of Medicine, University of Debrecen, Debrecen H-4012, Hungary; papa@med.unideb.hu (A.P.); ixchelt.cuaranta@med.unideb.hu (I.C.-M.); 2 Sanford Burnham Prebys Medical Discovery Institute at Lake Nona, Orlando, FL 32877, USA; mpeloquin@sbpdiscovery.org; 3 MTA-DE “Lendulet” Immunogenomics Research Group, University of Debrecen, Debrecen H-4012, Hungary

**Keywords:** PPARγ expression, human mutations, mouse models, metabolic syndrome, lipodystrophy, ligand activation

## Abstract

With the increasing number of patients affected with metabolic diseases such as type 2 diabetes, obesity, atherosclerosis and insulin resistance, academic researchers and pharmaceutical companies are eager to better understand metabolic syndrome and develop new drugs for its treatment. Many studies have focused on the nuclear receptor peroxisome proliferator-activated receptor gamma (PPARγ), which plays a crucial role in adipogenesis and lipid metabolism. These studies have been able to connect this transcription factor to several human metabolic diseases. Due to obvious limitations concerning experimentation in humans, animal models—mainly mouse models—have been generated to investigate the role of PPARγ in different tissues. This review focuses on the metabolic features of human and mouse PPARγ-related diseases and the utility of the mouse as a model.

## 1. Introduction

**Figure 1:**
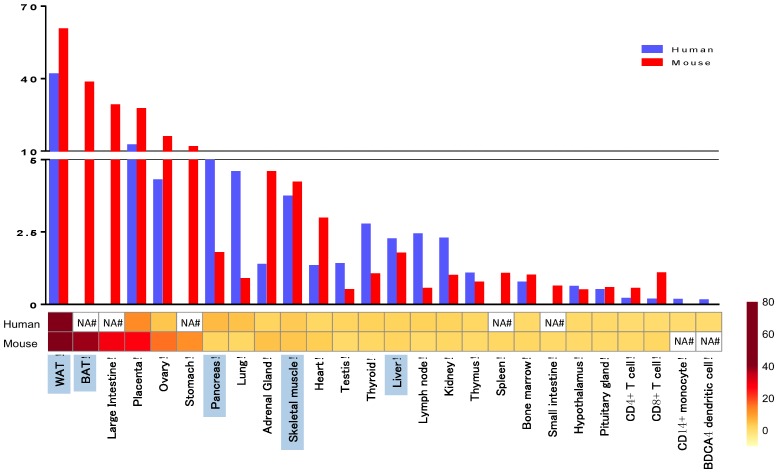
PPARγ tissue distribution in human and mouse. We re-analyzed the expression data set GDS596 (human) and GDS592 (mouse) from Su et al. [10] available on NCBI GEO database (http://www.ncbi.nlm.nih.gov/geo/query/acc.cgi?acc=GSE1133). The expression values were normalized to median and the tissues were selected based on the levels of PPARγ expression. Metabolic tissues are highlighted in blue. WAT = white adipose tissue, BAT = brown adipose tissue, NA = data not available.

**Figure 2:**
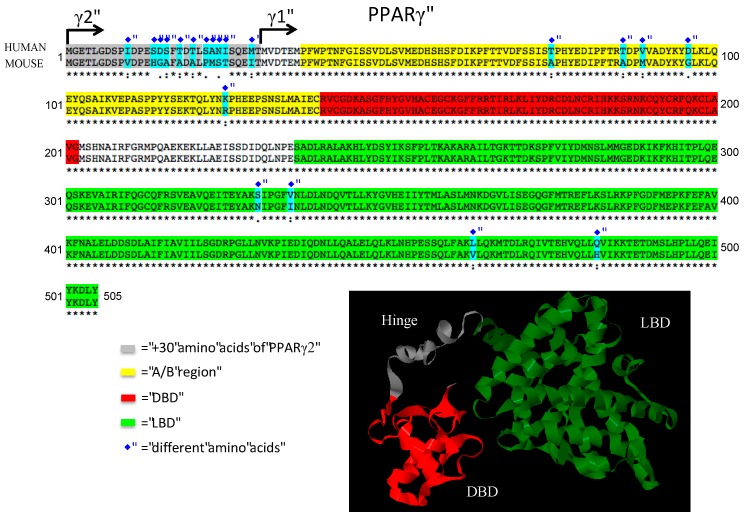
Comparison of PPARγ protein homology between human and mouse. We used Ensembl database to obtain the protein sequences and then we compared the human and mouse PPARγ sequences with Clustal 2.1 multiple sequence alignment program. For protein modeling of PPARγ Phyre2 web portal was used and for visualization Geneious 9.1.4 software was applied.

Peroxisome proliferator-activated receptors (PPARs) are ligand-inducible transcription factors of the nuclear receptor superfamily [1]. There are three PPARs in mammals: PPARα, PPARβ/δ and PPARγ. The PPARs forms obligatory heterodimers with retinoid X receptors (RXRs) and bind to PPAR-responsive elements (PPRE), which regulate the expression of different genes involved in adipogenesis, lipid metabolism and inflammation. PPARs have modular structures containing a N-terminal A/B region with a transactivation domain (AF1), a DNA binding domain (DBD) with two zinc-finger motifs and a C-terminal ligand-binding domain (LBD) with the ligand-dependent transactivation function (AF2) [2,3]. PPARγ was first identified in 1992 in Xenopus [4] and then in 1993 in mice [5]. PPARγ is highly expressed in white adipose tissue (WAT) and brown adipose tissue (BAT), where it plays a critical role in adipogenesis, lipid metabolism and insulin sensitivity. PPARγ is expressed at much lower levels in other metabolic tissues, such as liver and muscle but shows a relatively high expression in placenta, where it is a regulator of vascularization [6]. PPARγ is a modulator of lipid metabolism and inflammatory function in macrophages and dendritic cells [7,8,9]. Comparing the expression of PPARγ in human and mouse tissues, both show a very similar expression pattern, suggesting conserved function of PPARγ across species (Figure 1) [10]. PPARγ has two isoforms: PPARγ1 and PPARγ2. While PPARγ1 is expressed in many tissues, PPARγ2 is adipose tissue-specific under normal physiological conditions, however it is also expressed in macrophages [11]. These two isoforms differ at the N-terminal end of the protein, where PPARγ2 contains an additional 28 amino acids in humans and 30 amino acids in mouse that are absent in PPARγ1. The amino acid sequence of human and mouse PPARγ is highly conserved, with only nine amino acids are differing in the PPARγ1 (Figure 2). This suggests a very similar way of folding and DNA binding with RXR [12]. PPARγ can be modulated by posttranslational modifications such as ubiquitination, acetylation, phosphorylation and sumoylation. These modifications confer cell and tissue specificity [13,14].

Within the last two decades, PPARγ became a focus of attention as a transcription factor implicated in metabolic syndrome. Metabolic syndrome is a concerning public health issue worldwide, which is characterized by a cluster of different symptoms, including obesity, insulin resistance, hyperglycemia, hypertension, hypertriglyceridemia and decreased serum HDL cholesterol levels [15]. All of the aforementioned symptoms contribute to cardiovascular disease, the leading cause of death throughout the world. Researchers go to great lengths attempting to understand the human physiology and uncover those genetic, physiological and environmental changes, which contribute to impair metabolic processes. Plenty of studies have demonstrated the central role of PPARγ in metabolic diseases [16]. Thiazolidinediones (TZDs) are synthetic ligands and potent activators of PPARγ; they have been amply used in treating type 2 diabetes (T2D) in the past. Rosiglitazone and pioglitazone have been withdrawn from the US and European market due to critical cardiovascular diseases and bladder cancer as side effects [14,17,18]. Insulin resistance is a major player in the pathogenesis of metabolic syndrome. Furthermore, PPARγ agonists have been reported to modulate insulin sensitivity and glucose metabolism. Thus, it is of general research interest the finding of new PPARγ–modulators that could improve insulin sensitivity with less important side effects. However, clinical investigations involving human subjects have ethical and methodological limitations, creating a need for a physiologically relevant model organism. This need was addressed by using mice as a model organism for metabolic syndrome. Conversely, using model systems to investigate a biological process always raise the question: how good is the model that we use? In this review, we center our attention on the new developments of the field to answer this question.

## 2. Human Aspects of PPARγ in Metabolic Syndrome

### 2.1. PPARg Polymorphisms Related to Metabolic Traits without Lipodystrophy

**Figure 3:**
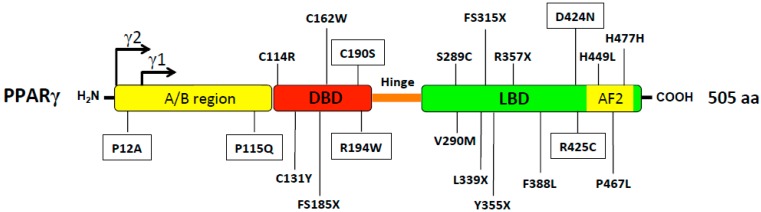
Human PPARγ mutations. Mutations on the PPARγ2 are marked with a black frame. A/B region = N terminal region with activation function 1; DBD = DNA binding domain; LBD = ligand binding domain; AF2 = activation function 2.

There are several mutations described in the PPARγ gene that affects metabolism traits in humans. These mutations have been classified previously as followed: common polymorphisms (Pro12Ala, His477His), dominant-negative (Val290Met, Cys162Tyr), haploinsufficient mutations (Arg425Cys, Phe388Leu), gain of function mutations (Pro115Gln) and promoter variants (P2 C-689T, P4 A-14G) [19]. In 1997, the most studied and well-characterized polymorphism in epidemiologic studies, Pro12Ala (rs1801282), was first described (Table 1). Susceptibility to T2D with the Pro12 allele and resistance with Ala12 allele was described later [20,21]. It has been hypothesized that the increased insulin clearance and sensitivity in Ala12 allele are due to improved lipolysis [22]. Furthermore, the association of LPL activity in vitro and in vivo has been reported [20,23]. In a large meta-analysis, the Pro12Ala SNP has been found to increase body mass index (BMI) [24]. The association of cardiovascular disease (CVD) and the Pro12Ala polymorphism has been widely studied. However, the results are contradictory. Ridker et al. found a protective role of the Ala allele for myocardial infarction risk [25]. However, in 2004 Tobin and colleagues did not find the same protective effect [26]. A more recent meta-analysis found an increased risk of CVD of the Ala allele in Caucasians patients but not in an Asian population [27]. Importantly, in genome-wide association studies (GWAS), the Pro12Ala variant was among those found in type 2 monogenic diabetes [28]. Moreover, this allele also interacts with BMI in regard to increasing insulin resistance [29]. Interestingly, PPARγ rs1801282 polymorphism has also been studied in several populations, and it exhibits population-based susceptibility to different metabolic traits. In overweight Brazilian pubertal sample, this polymorphism showed higher risks of altered insulin levels [30]. Meanwhile, Pro12Ala could predict BMI, overweight, and total cholesterol in females but not in male Taiwanese patients [31]. In children diagnosed with T2D the Pro12Ala polymorphism of PPARγ was significantly associated with obesity and T2D [32]. However, in a Japanese cohort, this polymorphism was not associated with BMI, and visceral and subcutaneous fat accumulation assessed by computed tomography [33]. Furthermore, Pro12Ala allele is a strong predictor for T2D susceptibility in Asian Indian Sikhs and Chinese population [34,35]. In Russian populations, this variant is associated with insulin sensitivity in type 2 diabetic and normoglycemic subjects [36]. PPARγ Pro12Ala polymorphism is associated with insulin sensitivity and BMI in patients with polycystic ovary syndrome (PCOS) [37]. Importantly, lifestyle interventions appeared to be allele-dependent. The association of Pro12 PPARγ carriers with T2D and low physical activity has been described [38]. PPARγ Pro12Ala variant improves glucose homeostasis as a result of regular exercising with a GWAS approach according to results from the HERITAGE Family Study [39]. Interaction of PPARγ Pro12Ala with dietary fat influences plasma lipids in subjects who are at risk for cardiometabolic diseases [40]. Furthermore, the PPARγ SNP rs1175544 influences the weight loss in a longitudinal study with short-term calorie restriction [37]. Importantly, Pro12Ala variant did not affect the response of pioglitazone treatment in patients with T2D (Figure 3) [41]. However, in a genome-wide study using ChIP-seq, RNA-seq and Gro-seq, Soccio et al. showed that PPARγ binding and the response to rosiglitazone depends on SNPs in human and mouse subcutaneous fat tissues and cell lines respectively [42].

The association between five PPARγ promoter variants and T2D has been described in T2D postmenopausal women [53]. In addition, different PPARγ polymorphisms (rs2972164, rs11128598, rs17793951, rs1151996, rs1175541, and rs3856806), contributed to the deterioration of β-cell function in Mexican Americans population with T2D risk [44]. In a large cohort of T2D cases and controls from multiple studies and ethnic groups, Majithia et al. in 2014 described unidentified PPARγ variants. Nine of these 49 variants have reduced activity in adipocyte differentiation and were associated with a higher risk of T2D [54] (Table 1).

Hypercholesterolemia and hypertriglyceridemia have also been associated with PPARγ polymorphisms in a large meta-analysis in 2012 by Asselbergs et al. [55]. Moreover, PPARα V162 allele increases total cholesterol and LDL-cholesterol levels. This effect was reduced by carrying the PPARγ T161 allele in patients with non-diabetic coronary heart disease (CHD) [45]. Another polymorphism associated with CHD is C161T in patients with T2D. The phenotype of this SNP was weakening with the presence of P12P homozygote genotype [46]. Moreover, in an Italian cohort, the 93695C > T PPARγ promoter polymorphism was found to have a protective role in acute coronary syndrome [56]. Furthermore, the C1431T PPARγ polymorphism was associated not only with altered plasma lipids during fasting but also with higher risk of an angiography defined CVD [47]. However, in a large meta-analysis there was no statistically significant difference of serum lipids levels in an Asian population carrying this SNP [57].

Epigenetic changes of the PPARγ gene locus have also been found in metabolic syndrome-related diseases. Recently Kokosar et al. investigate methylation and gene expression in adipose tissue in women with PCOS. Methylation and gene expression of PPARγ was inversely correlated in this study [58]. Furthermore, Nilsson et al. described differential DNA methylation in 15,627 sites, representing 7046 genes including PPARγ in adipose tissue from patients with T2D compared to control subjects [59].

PPARγ loss of function mutations have been reported in colorectal cancers. Not surprisingly in 2010 a novel germline mutation in this gene (S289C) was found in a patient with dyslipidemia, obesity, and hypertension not associated with T2D and a large intestine polyp that progressed to adenocarcinoma [48].

### 2.2. PPARg Mutations Associated with Lipodystrophy

Lipodystrophy is a syndrome characterized by adipose tissue deficiency; this results in ectopic lipid accumulation in organs and causes non-alcoholic fatty liver disease (NAFLD), reduced blood leptin insulin resistance and T2D [60,61]. Human lipodystrophies are genetic or acquired and may be partial or generalized. Familial partial lipodystrophies (FPLD) are diseases relating to abnormal adipose tissue topography and reduction in total fat mass. The FPLDs have been subclassified into three groups: FPLD1, FPLD2 or FPLD3. A set of mutations in PPARγ gene is associated with FPLD3 (Table 1). Patients with dominant-negative mutations in a single allele of PPARγ have partial lipodystrophy and insulin resistance. The FPLD3 clinical presentation is characterized by a deficiency of limb and gluteal fat, meanwhile abdominal and facial fat is usually preserved [62]. The presentation is usually in adulthood, but insulin resistance and lipodystrophy have been described in prepubertal children as well [63,64,65]. In the patients carrying PPARγ F388L mutant, the transcriptional levels of PPARγ were threefold lower than in the wild type in luciferase assay [64]. Two heterozygous mutations (P467L and V290M) were reported in the PPARγ ligand-binding domain and the clinical presentation in three patients was severe insulin resistance, liver steatosis, T2D and hypertension at an early age (Figure 3). Later, patients carrying these mutations were found to have partial lipodystrophy as assessed by a complete evaluation of body composition and fat distribution.

There have been approximately 60 patients in the world identified with FPLD3. The most recent reported mutations in the PPARγ gene that has been found in patients with FPLD3 are summarized below.

The PPARγ mutation D424N is located in the ligand-binding domain, and the patients carrying this mutation exhibited a loss of function; which is partially restored by adding the PPARγ agonist rosiglitazone during in vitro analysis using luciferase assays [66]. PPARγ H449L mutation was associated with hypertriglyceridemia, insulin resistance, and NAFLD in four patients related with variable severity in the clinical features. Three subjects presented diabetes or impaired glucose tolerance. Pioglitazone therapy in these three patients resulted in a modest improvement in their metabolic control and consistent menstrual cycles in the two female subjects [49]. Novel mutations in PPARγ (R165T and L339X) linked to FPLD3 are associated with a defective transrepression of cellular RAS leading to cellular dysfunction, contributing to the specific FPLD3-linked severe hypertension [50]. Recently, a heterozygous PPARγ mutation c.1040A > C was identified in all five patients of a family. The resulting amino acid substitution is predicted to disrupt critical molecular interactions at the ligand-binding domain [51]. All pathogenic mutations described until 2014 were heterozygous and located in the DNA- or ligand-binding domains of the PPARγ protein. Most of them show dominant negative activity [43,67]. Recently, Dyment et al. described a biallelic mutation at PPARγ that causes a congenital generalized lipodystrophy (E138V and R164W). A female patient presented a particular phenotype since birth: clear general absence of adipose tissue, later during childhood developed hypertriglyceridemia, pancreatitis, refractory diabetes, irregular menses and renal failure [52]. These new mutations open the possibility of analyzing PPARγ sequence in patients with congenital generalized lipodystrophy (CGL) when no mutation in well-established CGL causing genes could be found.

Further studies investigating PPARγ binding and general gene expression are needed in patients with partial lipodystrophies and human common polymorphisms. Also iPS technology should be used to the generation of patient-specific cell lines and the differentiation of such cells to adipocytes and other cell types should allow disease-in-a-dish type experiments and molecular dissection of the mutant receptor to cellular processes.

## 3. Mouse Models for Study the Role of PPARγ in Metabolic Diseases

### 3.1. PPARγ Full Body Knockout Mice

**Figure 4:**
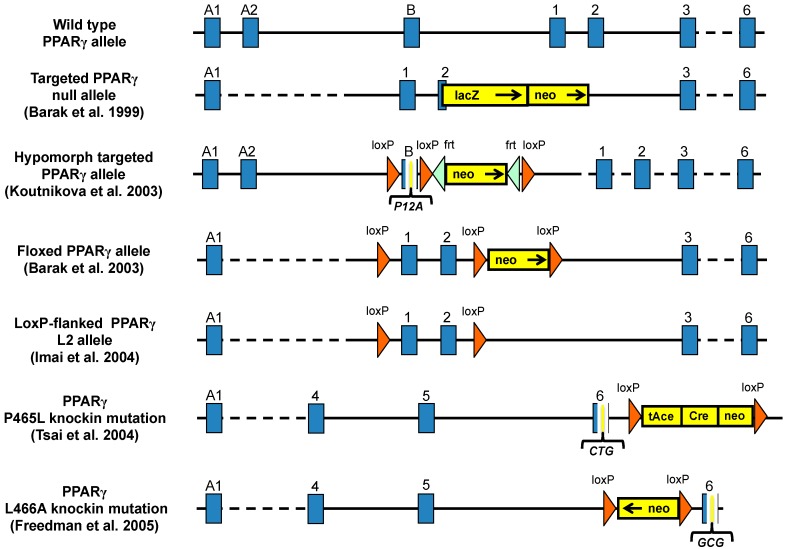
Gene editing strategies applied to the mouse PPARγ allele. The different targeting approaches that have been described in the literature are summarized in this figure.

The first attempts to generate whole body PPARγ knockout (KO) mice showed that loss of PPARγ caused impaired terminal differentiation of the trophoblast and placental vascularization resulting in utero lethality of null embryos tetraploid-rescued PPARγ-null mice survived and showed lack of adipose tissues, which established the essential role of PPARγ in adipogenesis (Figure 4) [68]. The solution for generating full body PPARγ null mice was the Mox2-Cre-floxed PPARγ (MORE-PG) KO, in which Cre recombinase is expressed only in epiblast-derived tissues and preserves PPARγ expression in the trophoblast but only 10% reach adulthood [69]. The characteristics of these mice are: lipodystrophy, organomegaly, decreased leptin and adiponectin in plasma, insulin resistance, elevated free fatty acids (FFAs) and hypotension (Table 2). Moreover, these mice show sex-dependent response to rosiglitazone, which induced regrowth of specific fat depots and improved insulin sensitivity in female, but not in male mice. In contrast, rosiglitazone improved glucose homeostasis with further increase in insulin production but not insulin sensitivity in male mice. Due to the high rate of mortality of this full PPARγ deletion, a tamoxifen inducible whole body PPARγ KO system has been used, and together with the MORE-PG mice, they showed a different gene expression of clock genes in relevant metabolic tissues than controls [70].

Expounding on these systems, Sox2Cre is another type of recombination technology for generating epiblast-specific conditional KO mice [71]. Sox2Cre-floxed PPARγ KO mice escape from embryonic lethality due to normal placental angiogenesis. Several diseases affect these full-body PPARγ deficient mice, therefore only some of them reach maturity [6]. The full body ablation of PPARγ using epiblast-specific KO mice gives an excellent opportunity to investigate the physiological effects of global PPARγ deletion in adult mice. The major limitation of the approach is that the pathologies affect multiple organs and therefore cell autonomous and primary effects are difficult to identify and dissect. A possible solution could be the development and more systematic usage of total body inducible and cell type specific inducible KO models in which the recombination can be induced at will in different developmental or diseases states.

### 3.2. Heterozygous PPARγ Mice

Mice heterozygous for PPARγ showed increased insulin sensitivity instead of the expected insulin resistance. These mice showed decreased triglyceride content in metabolic relevant organs due to elevated leptin expression and induction of fatty acid metabolism [72]. Heterozygous PPARγ mice are resistant to high fat diet (HFD) induced obesity and under these conditions, remained more sensitive to insulin than their WT counterparts [73]. This effect may be caused by the release of some genes that are repressed by PPARγ in adipose tissue. Although they use different mechanisms, activation and partial loss of PPARγ both increase insulin sensitivity [74]. Deletion of one PPARγ allele not only affected lipid storage, but mainly in fasting conditions, also reduced the expression of genes involved in glucose uptake and utilization, fatty acid synthesis, lipolysis and glycolysis. These deregulations led to reduce circulating adiponectin levels in the WAT. Expression of metabolic genes decreased in WAT, but was not affected in liver and skeletal muscle. In addition, there was a decrease in the metabolic rate and physical activity of the PPARγ^+/−^ mice, which was abolished by thiazolidinedione treatment, thereby linking regulation of the metabolic rate and physical activity to PPARγ [75].

### 3.3. Hypomorph Mouse Model

Targeting the exon B of adipose tissue specific PPARγ2 isoform generated the PPARγ KO in WAT (Figure 4). These mice also displayed decreased levels of PPARγ1 [76]. The homozygous (*PPARγ^hyp/hyp^*) mice are born normally, indicating that the PPARγ2 isoform may not be required for placental development. However, these animals present growth retardation, severe lipodystrophy and about a 40%–50% mortality rate before the age of five weeks. Neonatal *PPARγ^hyp/hyp^* mice have insulin resistance, hyperinsulinemia, hyperglycemia and fatty liver, which resembles to human CGL. In contrast, adult mice overcome the fatty liver and hyperlipidemia. However, the skeletal muscle and the heart accumulated more lipids and it was associated with glucose intolerance. The PPARγ agonist, rosiglitazone, reversed glucose intolerance, but not the insulin resistance in homozygote mice. Adipogenic markers and PPARγ target genes were reduced. The mild insulin resistance was explained by an up-regulation of β-oxidation in muscle. However, lipid metabolism and β-oxidation genes in the liver remained unchanged. This model demonstrates the compensatory mechanisms in the absence of WAT [76].

### 3.4. Ablation of PPARγ2 Isoform

Two PPARγ2 KO mouse models were generated [77]. PPARγ2 KO mice generated by Zhang et al. are viable, but have lipodystrophy and reduced leptin and adiponectin plasma levels. The PPARγ2 KO mice have insulin resistance in male but not in female mice. Surprisingly, the insulin resistance, hypertriglyceridemia and liver steatosis in these males could be reversed by PPARγ agonist treatment, demonstrating that PPARγ2 is not essential for TZDs action on insulin sensitivity [78]. Medina-Gomez et al. generated another PPARγ2 KO mouse model [79] that despite normal adipose tissue development, exhibit insulin resistance under chow diet, suggesting that PPARγ2 could modulate insulin sensitivity [79]. In both models in vitro adipocyte differentiation from precursors is impaired. This suggests a compensating mechanism, which protect in vivo adipogenesis. PPARγ2 deletion in the leptin deficient ob/ob background resulted in decreased fat mass, dyslipidemia, β-cell failure and insulin resistance. PPARγ2 isoform prevents lipotoxicity by promoting adipose tissue proliferation and decreasing ectopic lipid deposition in peripheral organs [80].

### 3.5. PPARγ Mutant Mice

Modeling the human PPARγ dominant negative mutations is important due to its impact in human metabolic diseases. Therefore, researchers generated mouse lines carrying similar dominant negative mutations in the PPARγ gene. Tsai et al. [81] generated a mouse model containing the P465L amino acid substitution in PPARγ (Figure 4), which is the equivalent with human (P467L) mutation [43]. The homozygous P465L PPARγ mutation is lethal, but the heterozygous animals display hypertension and altered adipose tissue distribution similarly to human phenotypes. In contrast with the severe insulin resistance in P467L PPARγ patients, the P465L PPARγ mutant mice have normal insulin sensitivity. However, P465L mutation shows more similarity to humans on obese *ob/ob* backgrounds [82]. Another dominant negative PPARγ (L466A) mouse model shows lipodystrophy, increased FFA levels, liver steatosis, hypertension and develops mild insulin resistance, when fed with high-fat diet (Figure 4) [83]. Moreover, mice harboring dominant negative mutations of PPARγ show altered adipose tissue localization and distribution revealing a role for PPARγ controlling the fat distribution in the body [81]. One of the models was the knockin of alanine at position 112 (S112A), which blocks the serine phosphorylation results in a constitutively active PPARγ, with elevated serum adiponectin and reduced FFA levels on high-fat diet. This result suggests that modulation of PPARγ phosphorylation may serve as pharmacological target for insulin sensitization [84].

Importantly, the well-known PPARγ2 P12A mutation in human populations was also generated in mice as a P12A knockin model. Homozygous Ala/Ala mice are viable, however they have lean phenotype, improved insulin sensitivity and plasma lipid profiles on chow diet. Heikkinen et al. demonstrates that P12A variant of PPARγ2 is an important modulator in metabolic control, but the effects depend on the metabolic context and gene–environment interactions [85].

Inducible PPARγ knockin mouse model was also developed in which the endogenous PPARγ gene was substituted with recombinant inducible PPARγ^ldi^ allele. The PPARγ^ldi/+^ mouse show reduced fat mass and insulin sensitivity giving a unique model of human conditional lipodystrophy [86].

### 3.6. Tissue Specific Ablation of PPARγ

#### 3.6.1. Adipose-Specific PPARγ Knockout

Imai et al. selectively deleted PPARγ in adipocytes of adult mice using the tamoxifen-dependent Cre-ERt2 recombination system (Figure 4). The mature PPARγ-null white and brown adipocytes die within a few days, demonstrating that PPARγ is essential for the in vivo survival of mature adipocytes. After some days without tamoxifen fat depots are replaced with newly formed PPARγ-positive adipocytes [87].

Two similar adipose-specific PPARγ KO mice have been published both KO mice use floxed PPARγ knock-in mice crossed with transgenic aP2-Cre mice model. He and colleagues report that WAT and BAT decreased in young mice, has decreased leptin and adiponectin plasma levels, increased circulating FFAs and triglycerides; therefore, developing liver steatosis. However, adipose-specific PPARγ KO mice have insulin resistance in adipose tissue and liver, but not in skeletal muscle when challenged with high-fat diet. Administration of TZDs to these mice improves insulin sensitivity in skeletal muscle and liver, but not in adipose tissue [88]. Jones et al. have published the other adipose-specific PPARγ KO mouse line. These animals exhibited impairment in brown and white adipogenesis and physiology. When fed with high-fat diet, these mice showed decreased weight gain despite hyperphagia, increased triglyceride levels, liver steatosis, reduced adiponectin and leptin levels and did not develop glucose intolerance or insulin resistance [89]. Characterization of in vivo glucose dynamics pointed to improved hepatic glucose metabolism as the basis for preventing high-fat diet-induced insulin resistance [89]. The differences between these rather similar models might be caused by the different expression of aP2-Cre. The aP2 promoter is a direct PPARγ target, such that PPARγ inactivation during differentiation will reduce the levels of Cre resulting different PPARγ inactivation and potentially distinct phenotypes. PPARγ activation by TZDs increases the uptake of fatty acids and the containing capacity of adipocytes. Selective activation of PPARγ in adipocytes can cause whole body insulin sensitization in mice without an increase of body weight [90]. Models of adipose tissue-specific impairment of PPARγ function demonstrate that PPARγ activity is necessary for normal adipose tissue development and maintenance.

Recently, Jonker and colleagues identified the fibroblast growth factor 1 (FGF1) as a critical transducer in the process of adipose tissue sensing nutrients and it stay under the regulation of PPARγ via the promoter of FGF1 gene. Interestingly, FGF1 KO have no significant phenotype under standard laboratory care, these mice develop severe diabetic phenotype and impaired adipose tissue expansion with multiple pathologies when challenged with a HFD. The phenotype of FGF1 KO mouse establishes the PPARγ–FGF1 axis as critical for maintaining metabolic homeostasis and insulin sensitization [91]. An in vivo conditional PPARγ KO adipocyte specific (Adipotrack marked cells) was described recently, and this model has been used to elucidate different cell progenitor depots and its importance in adipocyte differentiation within developmental and adult stages [92,93].

#### 3.6.2. Muscle-Specific Ablation of PPARγ

Skeletal muscle is one of the main insulin responsive tissues in the body. Although PPARγ is expressed to a much smaller extent in muscle than in adipose tissue (Figure 1), it is able to induce the expression of genes that regulate glucose uptake. Two independent groups examined mice with targeted PPARγ deletion in skeletal muscle using the creatinine kinase promoter driven Cre-loxP recombination system [94,95]. In the first study, Hevener et al. used older mice and showed that lack of PPARγ in skeletal muscle resulted in adiposity, severe insulin resistance, and being unable to respond to TZD treatment [94]. In another study, Norris et al. used younger mice with targeted deletion of PPARγ in muscle resulting in obese mice with no insulin resistance and remained responsive to TZD treatment [95]. The role of PPARγ in increasing lipid oxidation in muscle has been published [96]. Findings in muscle-specific PPARγ KO mice suggested that PPARγ in muscle can regulate whole-body lipid metabolism and insulin sensitivity, however TZDs have indirect and age dependent effects on skeletal muscle [14,77,95].

#### 3.6.3. Liver-Specific Disruption of PPARγ

PPARγ is expressed most highly in adipose tissue, but is also detectable in many other tissues such as liver (Figure 1), where PPARγ expression is increased in several mouse models of liver steatosis [97,98]. Liver PPARγ disruption has been developed using Cre recombination system driven by liver-specific albumin promoter [98]. Gavrilova et al. deleted PPARγ in the liver of A-ZIP/F-1 lipoatrophic mice. Lack of PPARγ in this lipoatrophic background protected the mice to develop fatty liver by reducing liver triglyceride and increasing serum FFA levels, but these mice have muscle insulin resistance. Liver-specific ablation of PPARγ in mice leads to increased adiposity and insulin resistance, but these mice respond to TZD treatment [98]. However, liver-specific PPARγ disruption on a lipoatrophic background results mice becoming resistant to TZD treatment, indicating that in the absence of WAT, the liver takes over the role of regulating lipid and glucose homeostasis [14,98]. Matsusue et al. also showed that disruption of liver PPARγ in leptin deficient *ob*/*ob* mice results decreased hepatic triglyceride accumulation, but elevated serum lipid levels and insulin resistance [99]. These reinforce the pivotal role of PPARγ in the liver regulating lipid homeostasis and protecting other organs from lipotoxicity and insulin resistance [77].

#### 3.6.4. PPARγ Ablation in Pancreatic Beta Cells

Pancreatic β-cells also express PPARγ (Figure 1) [100,101], where activation of PPARγ regulates the expression of genes involved in glucose-stimulated insulin secretion and TZDs can enhance the insulin secretion and insulin sensitivity in mice and human [102]. Surprisingly, deletion of PPARγ in mouse β-cells caused altered islet mass and morphology, but do not affect the whole body glucose homoeostasis. These mice showed weakened TZD response [103]. Another study used a pancreatic-specific PPARγ KO model generated by crossing mice with floxed PPARγ to mice with pdx-1 promoter driven Cre recombinase and showed that loss of PPARγ in the whole pancreas results normal size of β-cell islets, but hyperglycemia and impaired insulin secretion [104]. Vivas et al. shown that *ob/ob* mice with genetic ablation of PPARγ2, known as POKO mice failed to enlarged its β-cell mass. They identified genes that regulate β-cells proliferation and survival and identified some PPARγ dependent pathways (cholesterol biosynthesis, apoptosis through TGF-β signaling), which are differentially regulated in POKO mice [105]. However, Welters et al. detected minimal changes in gene expression of important β-cells genes in tamoxifen-inducible β-cell-specific PPARγ KO mice, which could be modified with HFD or rosiglitazone treatment. There were no significant differences in body weight, plasma insulin, glucagon and glucose levels when the mice are kept on normal diet. Based on this study PPARγ seems to be not directly essential for normal β-cell function [106].

#### 3.6.5. Disruption of PPARγ in Macrophages and Dendritic cells

PPARγ has an important role in many immune cell types [107]. Many studies have been focused on macrophages and dendritic cells. The PPARγ expression level in these cell types is low, but they have an important role in regulating expression levels of genes involved in lipid homeostasis and immune function [108,109]. Macrophages accumulated in adipose tissue in obese state are able to induce inflammation and affect glucose homeostasis [110]. Generation of inducible macrophage-specific PPARγ KO mouse revealed the importance of this receptor in the macrophages regulation of cholesterol efflux [111]. Macrophage-directed PPARγ KO mice are more predisposed to obesity and insulin resistance after challenged with HFD, however these mice do not have liver steatosis [112]. Macrophage PPARγ has been claimed to be required for normal skeletal muscle and liver insulin sensitivity and for the maturation of anti-inflammatory M2 type macrophages [113]. More recently it has been shown that STAT6 acts as a facilitating factor for PPARγ by promoting DNA binding and increasing number of genes connected to lipid metabolism and inflammatory response in macrophages and dendritic cells [114]. This work also established that M2 activation at the initial transcriptional response could take place without PPARγ. In addition PPARγ is a potent regulator of various processes in dendritic cells; however in vivo model system for DC-specific PPARγ ablation was unavailable for a long time. A recent study has shown that the dendritic cell-specific CD11c-Cre PPARγ^fl/fl^ conditional KO mice have spontaneous lung inflammation and emphysema. Using genome wide microarray analysis, they identified potential PPARγ regulated genes in emphysema [115]. Schneider and colleagues found that PPARγ is required for alveolar macrophage differentiation, however absence of PPARγ did not affect the development and recruitment of macrophages and dendritic cells in other tissues such as liver, brain, heart, kidneys, lamina propria and WAT. GM-CSF induces PPARγ expression in fetal monocytes and plays an important role in alveolar macrophage development. Transcriptome analysis of alveolar macrophage precursors from newborn mice showed that PPAR confers a unique alveolar macrophage signature and identity [116].

## 4. Testing of Novel PPARγ Modulators in Mice

Novel compounds have been developed in the last few years, which might be potential modulators of PPARγ. One of them is Z-551 that has both PPARα agonistic and PPARγ antagonistic activities. The effects of Z-551 were examined in wild type mice on HFD and it could suppress body weight gain, ameliorated insulin resistance and abnormal lipid metabolism, significantly reducing the plasma levels of glucose, FFAs, insulin and leptin [117]. Another potent modulator is a new thiazolidinedione, GQ-177, which has shown a therapeutic potential on diet-induced obesity and atherosclerosis. This molecule was identified as a partial and selective PPARγ agonist, which improved insulin sensitivity and lipid profile without affecting body weight, fat accumulation or bone density in LDLr-/- mice fed with high-fat diet [118].

## 5. Comparison of Human and Mouse Findings

Several years ago Heikkinen et al. [19] already summarized the role of PPARγ in human and mouse physiology listing the different human mutations found and mouse models generated until 2007. They highlighted the complex function of PPARγ in cell differentiation, inflammation, glucose and lipid homeostasis pointing ahead its role in metabolic diseases.

In this current review, we provide an update and focused on human and mouse experiments regarding metabolic syndrome, summarizing the earlier findings and more recent studies as well. As PPARγ effects occur in a tissue specific manner and the different PPARγ full agonists have severe side effects, also suggested the need of tissue selective PPARγ modulation. PPARγ allelic variants are the most common cause of metabolic traits related to the PPARγ gene. However, knockin mice (P465L) carrying a similar human mutation P467L have normal insulin sensitivity in contrast with the severe insulin resistance in these patients. This clearly indicates a difference in the response of the genetic variants between human and mouse. The Pro12Ala mutation of PPARγ2 is a risk factor of weight gains in human obese patients; in contrast, the Ala12 allele improves insulin sensitivity and has protective effect against obesity and type 2 diabetes mellitus in lean patients [20,119]. The Pro12Ala knockin mice show similar phenotypes, the Ala/Ala homozygous animals are leaner and more insulin sensitive than Pro/Pro mice on normal chow, however they put on weight and lost insulin sensitivity on HFD. It was suggested that the Pro12Ala variant is a diet-dependent metabolic sensor with the ability to modify the PPARγ2 efficacy [85]. To our current knowledge this polymorphism in human and in mouse behaves similarly. Although PPARγ binding is conserved in mouse and human orthologous regions, there is a marked difference in reclusion of this transcription factor due to the motif turnover in different species [120]. It is also important to point out that the PPARγ binding retention during mammalian evolution from mouse to human is C/EBPa interdependent [121]. Differences in PPARγ binding due to SNPs in mouse and human adipose tissue have been also reported [42]. Adding complexity to the system, polymorphisms in PPARγ cofactors can also affect insulin and glucose metabolism like PPARGC1A cofactor mutation Gly482Ser. These genes also should be considered T2D risk factors [122]. Phosphorylation of PPARγ at Ser273 by cyclin-dependent kinase 5 (Cdk5) can affect the expression of distinct PPARγ target genes increasing insulin resistance in mouse models. In obesity a variety of cytokines such as TNFα secreted by adipose tissue can induce the Cdk5 dependent PPARγ phosphorylation. Mutation of Ser273 to alanine and RSG could effectively block the Cdk5-mediated phosphorylation of PPARγ [123]. Interestingly, adipose tissue specific Cdk5 KO mice have increased PPARγ phosphorylation and insulin resistance due to ERK dependent phosphorylation [124]. Again underscoring the complexity and redundancy affecting PPARγ activation in vivo.

Mouse full body PPARγ KO MORE-PG does not mimic the human lipodystrophy findings (Table 2). Although, crossing these mice with the obese *ob/ob* model may provide some insight into the human lipodystrophy [98].

On the other hand, Gray et al. hypothesize that manipulations of PPARγ gene in mice generates very similar defects than in humans but these can only be seen when these mice are challenged with a HFD, exposure to low temperature, during exercise or food deprivation [82,125] arguing that external conditions are critical in the development of metabolic phenotypes and diseases in the presence of a particular genetic disposition.

Another important recent development is the discovery of PPARγ biallelic mutations in a human patient. These mutations are rare; therefore, fibroblasts and/or tissues derived from these patients are usually limited. Better full body PPARγ KO mice models are needed to elucidate PPARγ related CGL (Table 1 and Table 3).

In summary it is likely that a better and more strategic integration of mouse and human phenotypes and data will be possible by direct comparison of disease-in-a-dish type experiments on human derived iPS cell lines and mouse cellular models. As far as animal models are concerned more “dynamic” models allowing recombination of PPARγ in a temporal and tissue specific manner can accelerate the rate of discovery. CRISPR/Cas9 technology can be used to *knock out* or *knock in* genes in whole body animal models and in human cell lines; allowing researchers to study at molecular and physiological level the effect of this gene disruption. This can also lead to identification of the tissue and cell type specific roles including tissue-specific gene expression. The extensive usage of genomic and epigenomic approaches are also going to help dissect the gene expression networks coordinated by the receptors. The ultimate goal of therapy should be to develop tissue selective PPARγ modulators to avoid side effects. For this to happen, mouse and human need to go forward hand in hand in an even more intertwined manner.

## 6. Conclusions

Mice have many genes in common with humans (99% of human genes are conserved in mouse genome) and show many similarities in organ physiology, metabolic processes and pathogenesis of different diseases. Mice are excellent model organisms for other reasons as well. First of all, they are small in size and have a short generation time, which makes breeding and housing relatively simple and cost-effective. Second, since the mouse genome is known, the use of genetically modified mouse models in research and preclinical studies has increased. Furthermore, the mouse is the only mammalian model in which it is technically possible to replace a particular mouse gene with its human counterpart. These so-called “humanized” mouse models are able to produce the human version of the protein of interest, or it can be created to carry a mutated version of the human gene, which is known to be associated with a human disease.

Comparing the findings from human and mouse PPARγ related metabolic diseases, we can conclude that mice models can generally be used to investigate and more deeply understand the processes of human diseases. However, it is important to know that despite genetic and physiological similarities, mice have a lot of specific features, which make it difficult to extrapolate mouse results to human. Moreover, there are different conditions in mice, such as genetic background, gender, age, diet and environmental conditions, which could further modify the results. In the last decade, genome-wide studies have changed the epidemiological and functional research of PPAR variants in both human and mouse. However, more systemically used epigenetic and transcriptomic analyses are necessary in the different PPARγ mouse mutants for elucidating PPARγ and its cistrome’s role in metabolic syndrome. The future of PPARγ research relies on using humanized mouse models coupled with human iPS cells derived tissues and genome-wide studies for not only clarify the molecular mechanism of PPARγ in its target genes that have an impact on metabolic syndrome conditions, but also to find suitable PPARγ modulators for human insulin resistance and diabetes treatment.

## Figures and Tables

**Table 1:** Summary of human PPARγ polymorphism associated to metabolic syndrome conditions.

Polymorphism	Metabolic Traits Involved	References
Pro12Ala	T2D	[20,32]
	Monogenic diabetes	[21]
	Higher BMI	[24,32]
	Altered insulin levels	[30]
	Insulin sensitivity	[36]
	BMI and insulin sensitivity in PCOS	[37]
P467L V290M	Insulin resistance, liver steatosis, T2D and hypertension	[43]
Promoter variants polymorphism rs29722164 rs11128598 rs17793951 rs1151996 rs1175541 rs3856806	Deterioration of B-cell function	[44]
V162	Increase total cholesterol and LDL-cholesterol levels	[45]
C161T	CHD in patients with T2D	[46]
C1431T	Altered fasting serum lipids and risk factor for CHD	[47]
S289C	Dyslipidemia, obesity and hypertension	[48]
H449L	Hypertriglyceridemia, insulin resistance and hepatic steatosis, FPLD3	[49]
R165T L339X	FPLD3 and severe hypertension	[50]
c.1040A > C	FPLD3, Diabetes Mellitus, hypertension and dyslipidemia	[51]
Biallelic mutation E138V and R164W	CGL, hypertriglyceridemia, diabetes mellitus, pancreatitis and renal failure	[52]

T2D = type 2 diabetes mellitus; BMI = body mass index; PCOS = polycystic ovarian syndrome; LDL = low- density lipoprotein; CHD = coronary heart disease; FPLD3 = familiar partial lipodystrophy 3; CGL = congenital general lipodystrophy.

**Table 2:** Comparison of the metabolic features of PPARγ whole body and tissue-specific KO mice.

Features	Mouse Models								
	MORE- PG KO [69]	HET-PPARγ [72,75]	HYPO- PPARγ [76]	PPARγ2 KO [78]	Adipo PPARγ KO [88,89]	Sc.M. PPARγ KO [94,95]	Liver PPARγ KO [98]	β-cell PPARγ KO [103]	MΦ PPARγ KO [112]
Obesity	No	↓	↓	No	↓ (HFD)	↑ (HFD)	No	No	↑ (HFD)
Insuline resistance	Yes	IS	Yes	Yes (male)	unclear	Yes	Yes	No	Yes
Glucose tolerance	↓ (male)	ND	↓	↓	ND	↓	ND	NC	↓ (HFD)
Type 2 diabetes	Yes (male)	No	ND	ND	Yes	Yes	ND	No	ND
Lipodystrophy	Yes	No	Yes	Yes	Yes	No	No	ND	ND
Liver steatosis	No	No	No	No	Yes	ND	No	ND	No
Hypertension	hypoten.	ND	ND	ND	ND	ND	ND	ND	ND
Organomegaly	Yes	No	No	No	ND	Yes	No	ND	No
Food intake	NC	↓	NC	NC	↑ (HFD)	↓	NC	ND	ND
Triglicerides	↑	↓	↓	NC	↑	↑	↑ *	ND	NC
Free fatty acids	↑	↓	↑ (fed)	ND	↑	↑	NC	ND	ND
Cholesterol	ND	ND	ND	ND	ND	ND	NC	ND	LDL ↓
Glucose	↑	ND	↑ (fed)	NC	NC	↑	↑ *	NC	↑ (HFD)
Insulin	↑	↓	↑	ND	↑	↑	↑ *	NC	↑ (HFD)
Leptin	↓	↑	↓	↓	↓	↑	↑ *	ND	↑
Adiponectin	↓	↑	↓	↓	↓	ND	↓ *	ND	↓
TZD effectiveness	ND	Yes	ND	Yes	partial	partial	Yes	Yes	Yes

HET = heterozygous; HYPO = hypomorph; Adipo = adipocyte; Sc. M. = skeletal muscle; MΦ = macrophage; HFD = on high fat diet; IS = insulin sensitivity; hypoten = hypotension; male = just in male mice; fed = in fed state; * = only in 40 weeks old mice; NC = not changed; ND = not determined.

**Table 3:** Comparison of the metabolic features between human and mouse PPARγ mutants.

Features	Human Mutants				Mouse Mutants	
	P12A Mutant [20]	P467L Mutant [43,65]	F388L Mutant [64]	Biallelic E138V R164W [52]	P12A Mutant [85]	P465L Mutant [81]
Obesity	Yes	No	No	No	No	No
Insuline resistance	Yes	Yes	Yes	Yes	IS	No
Glucose tolerance	ND	↓	ND	ND	↑	↑
Type 2 diabetes	Yes	Yes	Yes	Yes	No	No
Lipodystrophy	No	No	FPLD3	CGL	No	redistr.
Liver steatosis	ND	ND	No	ND	ND	ND
Hypertension	ND	Yes	Yes	No	ND	Yes
Organomegaly	No	ND	No	Yes	No	ND
Food intake	ND	ND	ND	ND	NC	NC
Triglicerides	↑	↑	↑	↑	↓	NC
Free fatty acids	ND	ND	ND	ND	NC	NC
Cholesterol	↑	HDL ↓	HDL ↓	NC	↓	NC
Glucose	↑	ND	↑	↑	NC	NC
Insulin	ND	↑	↑	↑	NC	↑ (HFD)
Leptin	ND	ND	ND	↓	NC	ND
Adiponectin	ND	ND	ND	↓	NC	ND
TZD effectiveness	ND	ND	partial	ND	partial	ND

FPLD3 = familiar partial lipodystrophy 3; CGL = congenital generalized lipodystrophy; HFD = on high fat diet; HDL = high-density lipoprotein; IS = insulin sensitivity; NC = not changed; redistr. = redistribution of adipose tissue; ND = not determined.
